# Impact of a four-domain intrinsic capacity measure on falls: findings from the EPOSA study

**DOI:** 10.3389/fragi.2025.1645712

**Published:** 2025-09-05

**Authors:** Chiara Ceolin, Paola Siviero, Federica Limongi, Marianna Noale, Giuseppe Sergi, Stefania Maggi, the EPOSA Research Group

**Affiliations:** ^1^ Geriatric Unit, Department of Medicine (DIMED), University of Padova, Padova, Italy; ^2^ Department of Neurobiology, Care Sciences and Society, Aging Research Center, Karolinska Institutet and Stockholm University, Stockholm, Sweden; ^3^ Institute of Neuroscience, National Research Council, Padova, Italy

**Keywords:** intrinsic capacity, falls, ageing, EPOSA, epidemiology

## Abstract

**Objective:**

Low Intrinsic Capacity (IC) has been associated with adverse health outcomes in older adults, including falls. This study examines a four-domain measure of IC (cognition, psychological, locomotion, vitality) and its relationship with fall risk in a large European cohort.

**Methods:**

Data were from the European Project on Osteoarthritis (EPOSA) study. IC, operationalized using four domains (cognition, psychological, locomotion, vitality), was assessed on 2,597 adults (65–85 years) of six countries, with follow-up data at 12–18 months. Logistic regression models were used to evaluate its association with falls after the baseline, adjusting for sociodemographic, socioeconomic, and health factors, including osteoarthritis.

**Results:**

The median IC score was 6 (IQR: 5–7) and varied by country. Italy, Spain and the United Kingdom had the lowest scores, particularly in the locomotion, psychological and cognitive domains. Of the 2,127 participants who completed the follow-up, 26.8% reported falls. Multivariable analysis revealed significant associations between falls and IC, joint replacement, clinical osteoarthritis, analgesic/anti-inflammatory medication use and tobacco consumption, as well as a borderline association with cardiovascular disease. Individuals with a low IC score (<5) had a 1.57 times greater risk of falling.

**Discussion:**

Low IC, based on four domains, predicts falls in older adults. Identifying individuals with low IC can aids targeted interventions to reduce risk and health burdens. Prevention programs should integrate physical, cognitive, and psychological support while considering clinical and demographic interactions. These findings highlight the value of multidomain IC assessment as a tool for promoting healthy aging.

## 1 Introduction

Intrinsic Capacity (IC) is defined by the World Health Organization (WHO) as ‘the composite of all the physical and mental capacities that an individual can draw on’ at any given time (Organization, 2025). Research has consistently shown that reduced IC is associated with several adverse outcomes in older adults, such as functional decline and increased mortality risk (Sánchez-Sánchez et al., 2024). Furthermore, frailty—a condition often characterized by low IC—has been linked to a higher risk of falls (Cheng and Chang, 2017), suggesting that diminished IC may be a key underlying factor in fall susceptibility.

Falls themselves represent one of the primary challenges in geriatrics due to their significant impact on health, autonomy, and quality of life (Biasin et al., 2023). These events not only cause immediate physical injuries—such as fractures (Shehu et al., 2023)—but also lead to a cascade of functional and psychological decline that further compromises independence (Drahota et al., 2024). Considering the complex interplay of biological, psychological, and social factors that contribute to fall risk (Smith et al., 2023), it is plausible to hypothesize—given the strong association between frailty and falls (Yang et al., 2023)—that IC is closely linked to the risk of falls.

Recent studies have provided consistent quantitative evidence linking lower IC to a higher risk of falls. In community-dwelling octogenarians from the IlSIRENTE study, higher IC was associated with substantially lower odds of recent falls (OR 0.33, 95% CI: 0.16–0.82), with the locomotion domain independently associated with fall risk (OR 0.98, 95% CI: 0.96–0.99) (Cacciatore et al., 2024). Similarly, in a large Indian cohort of more than 24,000 older adults, those with high IC had a lower prevalence of falls (9.42% vs. 13.34%) and significantly reduced odds of fall-related outcomes, including multiple falls (OR: 0.73, 95% CI: 0.58–0.96) (Muneera et al., 2023). More recently, a one-year longitudinal study in Chinese community-dwelling older adults found that declines in locomotive (OR = 25.87), psychological (OR = 25.29), and sensory (OR = 10.75) domains strongly predicted falls, while cognitive decline predicted disability (Liu et al., 2025). Collectively, these findings highlight the importance of assessing multiple IC domains to identify high-risk individuals and guide targeted preventive strategies.

In light of these considerations, this study utilizes data from the European Project on Osteoarthritis (EPOSA) study, a large European cohort study with a 12–18-month follow-up, to examine the association between IC and falls after baseline. The analysis accounts for sociodemographic, socioeconomic, and health factors, including osteoarthritis (OA), among older adults across six countries.

## 2 Materials and methods

### 2.1 Study design and population

Participants are from the EPOSA cohort, which includes random samples from five existing population-based cohorts studies (Germany, the Netherlands, Spain, Sweden, and the United Kingdom), as well as a newly recruited sample from Italy (van der Pas et al., 2013). EPOSA was a population-based study of 2,942 adults between the ages of 65–85 years old, resident in six European countries (Germany, Italy, the Netherlands, Spain, Sweden, and the United Kingdom). After having written informed consent, all participants underwent a baseline clinical examination and interview at home or in a healthcare center by trained researchers, between November 2010, and November 2011, and a follow-up interview 12–18 months later. The local research ethics committees approved the study (Germany: Universitat Ulm Ethikkommission [312/08]. Italy: Comitato Etico Provinciale Treviso [XLIV-RSA/AULSS7]. Netherlands: Medisch Ethische Toetsingscommissie Vrije Universiteit Amsterdam [2002/141]. Spain: Comité Ético de Investigación Clínica del Hospital Universitario La Paz Madrid [PI-1080]. Sweden: Till forskningsetikkommittén vid Karolinska Instituted Stockholm [00–132]. United Kingdom: Hertfordshire Research Ethics Committee [10/H0311/59]).

### 2.2 Intrinsic capacity

In this study, IC was operationalized using four domains available in the EPOSA dataset: cognition, psychological, locomotion, and vitality. The sensory domain (vision and hearing), part of the WHO construct, was not available. Each domain was scored from 0 to 2, with 0 or 1 indicating a worse status and 2 indicating a better one. According to previous studies (Muneera et al., 2023; Ma et al., 2021), a composite IC score was then calculated, ranging from 0 to 8, with lower scores representing worse status.

1. For the cognition, the Mini-Mental State Examination (MMSE) was used (Folstein et al., 1975), a widely validated tool for cognitive impairment, with good internal consistency (Tombaugh and McIntyre, 1992). The total score ranging from 0 to 30 (with lower score indicating greater cognitive impairment) was classified as 0 for scores ≤18, 1 for scores between 18–24, 2 for scores ≥24. MMSE scores were not corrected for age or educational level, factors that may influence cognitive performance but which were considered in the analysis.
2. The psychological dimension was assessed using the Hospital Anxiety and Depression Scale (HADS) (Zigmond and Snaith, 1983), which comprises separate subscales for anxiety and depression. Previous studies have demonstrated the high internal consistency of both subscales (Bjelland et al., 2002). Scoring was defined as follows: 1 point for subscale scores <8, 0.5 points for scores between 8–10, 0 point for scores ≥11, with higher scores indicating a greater presence of symptoms.
3. The locomotion dimension was assessed with three tests (Guralnik et al., 1989): walking speed, repeated chair stands and standing balance. These tests are adapted from the Short Physical Performance Battery (SPPB) and are validated objective measures of lower extremity function and mobility, commonly used in geriatric populations (Guralnik et al., 1994). As performance-based tests, they are not evaluated through internal consistency metrics. Walking speed was measured by the time taken to walk 3 m ‘as fast as possible but not running’. Chair stands were measured by the time taken to rise from a chair five times in normal time, without using the hands. The standing balance test was measured by the ability to perform the tandem stand for 10 s (with one foot behind the other and the heel of the first foot directly touching the toes of the other foot). The participants’ times for walking speed and repeated chair stands were divided into country-specific quartiles (scores 1–4, participants who were unable to perform these two tests were scored 0). The tandem stand test was categorized into three groups. For comparability with the other tests, these three groups received the following scores: unable (<3 s = 0), able to hold position for 3 to <10 s (Sánchez-Sánchez et al., 2024), and able to hold position for 10 s (Biasin et al., 2023). Each test (walking speed, chair stand, and balance) was scored from 0 to 4, with a score of 0 representing inability to carry out the test, and 4 the best performance. A total physical performance (PP) score was obtained by summing the scores of each test, ranging from 0 to 12 (Guralnik et al., 1994). Locomotion was recorded as 0, for a summary performance score ≤6, 1, for a summary performance score between 7-9, and 2, for a summary performance score ≥10.
4. Vitality was measured by body mass index (BMI) and the grip strength (Roberts et al., 2011), which are objective indicators of nutritional status and muscle strength, respectively, approved by international experts (Bautmans et al., 2022). The BMI, which refers to the weight in kilograms (measured with a calibrate scale) divided by height in meter squared (kg/m2, measured with a stadiometer), is considered as an indicator of the balance between energy intake and energy expenditure, and a lower BMI suggests an increased risk of malnutrition. The BMI levels have been classified according to the WHO classifications: underweight ≤18.4; normal = 18.5 to 24.9; overweight = 25.0 to 29.9; obese ≥30.0. Grip strength was measured with a strain–gauged dynamometer, considering the mean of two right- and left-hand maximum measurements or the maximum value if only one hand could be used. The vitality score was obtained by adding the scores of BMI classified as 0 for lower BMI/underweight or obese, 0.5 for higher BMI/overweight weight, and 1 for normal, and the grip strength scores, classified considering the tertiles: 0 for values ≤ 22, 0.5 for values between 22–32, 1 for values > 32.

### 2.3 Outcome

The primary outcome of the study was fallen in the last 12–18 months, self-reported using the question, ‘Did you fall in the past year?’ coded as ‘no’ and ‘yes’.

### 2.4 Baseline characteristics

The baseline characteristics considered included age, sex, country of residence, education level, marital status, income, alcohol and tobacco consumption, chronic diseases, medications being taken, joint replacements and OA.

Educational level was categorized as up to elementary education versus higher levels of education. Marital status was categorized as being single or never married, divorced, widowed, living apart versus married or cohabitating, or in a registered partnership. A monthly income capable of making ends meet was classified as ‘only with great difficulty’/’with some difficulty’, ‘fairly easily’ and ‘easily’. Self-reported information about tobacco consumption was classified as ‘currently’, ‘in the past’ vs. ‘never’, while those on alcohol consumption ‘yes’ versus ‘no’. Self-reported presence of chronic conditions referred to: non-specific lung disease (i.e., asthma, chronic bronchitis or pulmonary emphysema, etc.), cardiovascular disease (i.e., cardiac valve disease, coronary heart diseases, arrhythmia, pacemaker, cardiac arrest, etc.), peripheral artery disease, diabetes mellitus, stroke, cancer and osteoporosis, lasting at least 3 months or which caused the individual to seek a physician’s attention (each dichotomized as present versus absent). Among the medication used over the past 2 weeks were analgesics and/or anti-inflammatory drugs and psycholeptics drugs; these were categorized as either medication use versus non-use. The presence of previous joint replacements was assessed by asking participants if they had ever had joint replacement surgery. If the response was affirmative, the participant was asked about the location and the reason for the joint replacement; the variable was classified as ‘lower limb’, ‘other joints’ versus ‘no’.

The clinical diagnosis of OA was determined on the basis of the participant’s medical history and a physical examination, according the clinical criteria of the American College of Rheumatology (ACR) (Altman, 1991), and the European League Against Rheumatism (Zhang et al., 2009). Clinical hand OA was diagnosed using specific sections of the Australian Canadian Osteoarthritis Hand Index (AUSCAN) (Bellamy et al., 2002). Clinical hip/knee OA, defined as the presence of OA in at least one or both of these joints, was diagnosed using specific sections of the Western Ontario and McMaster Universities Arthritis Index (WOMAC) examining pain and stiffness (Bellamy et al., 1988; Roorda et al., 2004). Pain in the hip/knee on at least one side was also evaluated during the physical examination. As far as clinical OA was concerned, the participants were classified as: (Organization, 2025) no OA (Sánchez-Sánchez et al., 2024), only hand OA (Cheng and Chang, 2017), hip and/or knee OA (Biasin et al., 2023), hip and/or knee OA combined with hand OA.

### 2.5 Statistical analysis

Only participants with complete data for all the variables were included in the analyses. Weights based on sex and five-year age categories according to the 2010 Standard European Population, calculated for each individual within each country of the EPOSA study (van der Pas et al., 2013) were used only in the descriptive analyses. Categorical variables were reported as proportions, and continuous variables as means and standard deviations (SD), and medians with interquartile ranges (IQRs). Normal distributions of continuous variables were tested using the Kolmogorov–Smirnov test. Significant differences between countries or groups of participants were evaluated using the Kruskal–Wallis test, the Wilcoxon rank-sum test, or the χ2 test, as appropriate.

The predictors of falls were assessed using logistic regression models adjusted for sex, age, and country. Each independent variable was examined to check the appropriate categories if categorical and the linearity in the logit and the scale if continuous. A multivariable model containing all the variables was fitted using a stepwise selection procedure (P to enter = 0.15 and P to remain = 0.10) to select them. All the interactions between the variables in the final model were checked; interaction terms with P ≤ 0.10 were retained in the final model. Odds ratios (ORs) were presented with their 95% confidence intervals (CIs).

Tests were two-tailed, and P < 0.05 was considered statistically significant. Analyses were performed using SAS software (SAS Institute Inc, Cary, NC), version 9.4.

## 3 Results

Of the 2,942 individuals initially enrolled in the EPOSA study, 2,597 (86.6%) had complete baseline data, and 2,127 had follow-up data available for all variables included in the analyses.

Compared to participants who completed the follow-up (n = 2,127), those who dropped out (n = 470) were significantly older, more likely to be female, predominantly residing in Sweden and the United Kingdom, and had a lower income (Supplementary Table S1). IC data are reported in Supplementary Table S2.

### 3.1 Baseline characteristics and intrinsic capacity

The 2,597 participants with complete, country-stratified (weighted) data had a median age of 73 years (IQR: 70–78, range: 65–85 years). Women comprised 51.3% of the sample, and nearly 30% had a diagnosis of OA. Additionally, 11% had undergone a joint replacement, approximately 25% reported cardiovascular disease and the use of analgesic/anti-inflammatory medications, 10% took psycholeptic medications and more than 50% had an income sufficient to make ends meet. Alcohol consumption was reported by 76% of participants, while around 8% used tobacco. Significant differences were observed between countries (Supplementary Table S3).

Overall, the median IC score was 6 (IQR: 5–7) (Supplementary Table S4). More than half of the participants exhibited some degree of impairment in the locomotion and vitality domains. Median IC scores were lower in Italy, Spain, and the United Kingdom, while Sweden, Germany, and The Netherland had the highest ones. Specifically, Italy showed greater impairment in locomotion, psychological, and cognitive domains; Spain in vitality, psychological, and cognitive domains; and the United Kingdom in vitality, locomotion, and cognitive domains.

**FIGURE 1:**
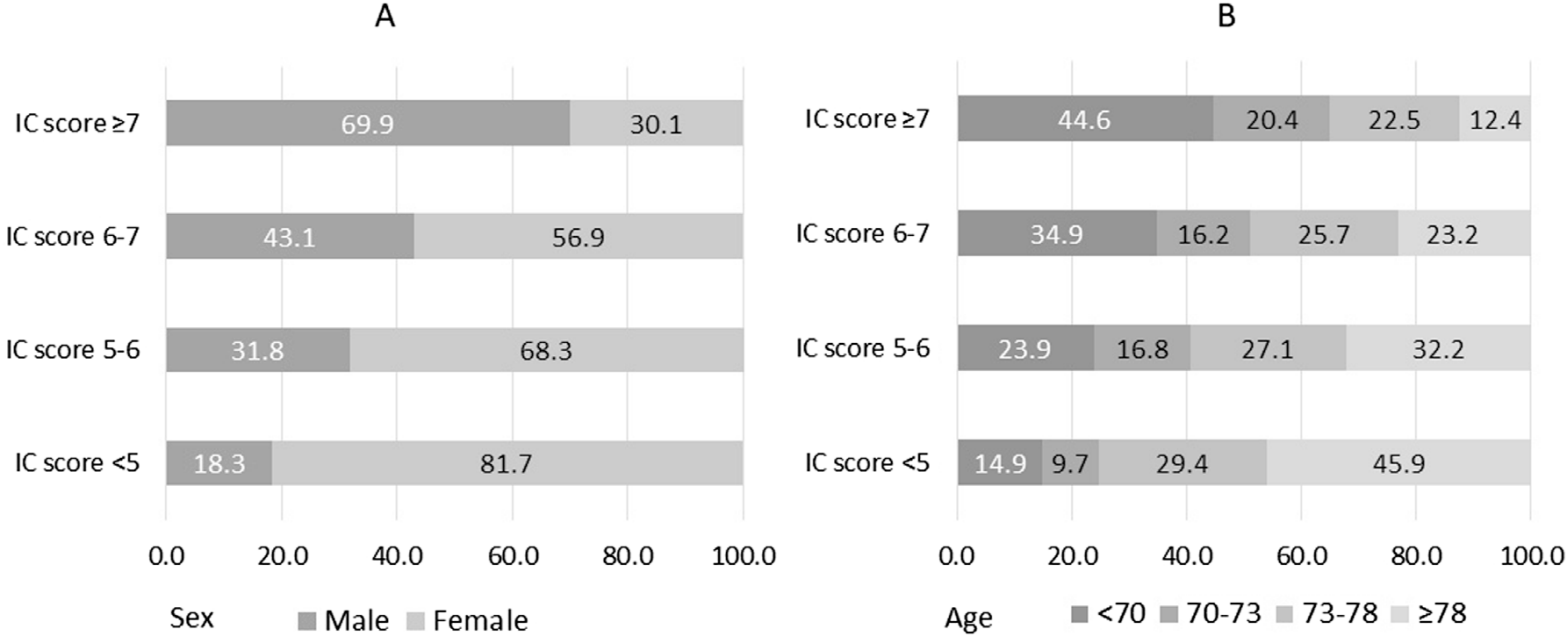
Sex **(A)** and age **(B)** according to quartiles of intrinsic capacity (IC). IC possible scores range from 0 to 8, with 0 indicating worst status. Age is classified considering the quartiles.

Sex and age differences across IC quartiles were significant (Figure 1). In the group with lower IC scores, 81.7% were women, and 45.9% were older than 78 years. In contrast, in the group with higher IC scores, these proportions were notably lower, at 30.1% and 12.4%, respectively.

### 3.2 Intrinsic capacity and falls

After 12–18 months, 569 out of 2,127 participants (26.8%) reported experiencing a fall (Table 1). Individuals who fell differed significantly from those who did not in several ways: they were more likely to be female, older, living in Sweden, Spain, or Germany, and single, divorced, widowed, or living alone. Falls were also more common among individuals who did not use tobacco, had OA, had undergone joint replacements in the lower limbs, and used analgesic or anti-inflammatory medications.

**TABLE 1:** Baseline characteristics for falls at 12–18 months Follow-up.

Baseline characteristics		12–18 months follow-up		*P*
	Total (n = 2,127)	No fall (n = 1,558)	Fall (n = 569)	
Age, mean ± SD, median (IQR), years	73.7 ± 5.0)	73.6 ± 4.9	74.2 ± 5.1	0.0240
	73 (70–77	73 (70–77)	74 (70–78)	
Female sex, %	50.0	47.9	55.7	0.0014
Country, %				0.0267
Germany	13.7	13.0	15.6	
Italy	15.0	15.0	15.0	
Netherlands	18.2	19.5	14.8	
Spain	19.1	19.9	17.1	
Sweden	20.2	18.9	23.7	
United Kingdom	13.7	13.7	13.8	
Up to elementary education, %	42.5	42.8	41.5	0.6010
Marital status (Single/divorced/widowed/living apart), %	33.3	31.7	37.7	0.0122
Income, %				0.5838
With great/some difficulty	16.6	16.1	18.0	
Fairly easily	50.3	50.7	49.3	
Easily	33.1	33.3	32.7	
Alcohol consumption (yes), %	77.3	78.1	74.9	0.1229
Tobacco consumption, %				0.0053
Never	49.5	48.2	53.2	
Currently	7.6	8.7	4.7	
In the past	42.9	43.1	42.1	
Chronic lung disease, %	12.7	12.1	14.3	0.1860
Cardiovascular disease, %	24.0	23.0	26.7	0.0831
Peripheral artery disease, %	9.9	9.8	10.1	0.8232
Diabetes mellitus, %	11.8	11.6	12.3	0.7068
Stroke, %	4.4	4.4	4.5	0.9965
Cancer, %	14.1	13.8	15.1	0.4268
Osteoporosis, %	15.3	14.4	17.6	0.0728
Clinical osteoarthritis, %				0.0004
No	70.5	72.4	65.6	
Hand	8.4	7.3	11.1	
Hip and/or knee	13.3	13.6	12.5	
Hand and (hip and/or knee)	7.8	6.7	10.8	
Joint replacements, %				0.0004
No	88.9	90.1	85.6	
Lower limb	9.6	8.2	13.6	
Other	1.5	1.7	0.8	
Analgesic/Anti-inflammatory medication, %	24.2	22.3	29.5	0.0008
Psycholeptic medication, %	8.8	8.4	10.0	0.2577

Weighted data except numbers of participants, age, and sex. SD, standard deviation; IQR, interquartile.

Regarding IC and its domains (Table 2), participants who experienced falls exhibited greater cognitive deficits and more pronounced depressive symptoms, indicating a worse psychological domain. They also had lower strength and, even after accounting for BMI, reduced vitality. Locomotion was particularly impaired, as reflected in slower gait and chair test performances, as well as an overall lower PP score. Consequently, their IC scores were low.

**TABLE 2:** Intrinsic Capacity domains for falls at 12–18 months Follow-up.

Intrinsic capacity domains			12–18 months follow-up		*P*
		Total (n = 2,127)	No fall (n = 1,558)	Fall (n = 569)	
Cognition					
Cognition (MMSE^a^), %	0 (MMSE score ≤18)	0.5	0.5	0.3	0.0010
1 (MMSE score 18–24)		5.8	4.6	8.9	
2 (MMSE score ≥24)		93.8	94.9	90.8	
Psychology					
Anxiety symptoms (HADS^b^), %	0 (HADS <8)	6.2	5.9	7.0	0.1191
	0.5 (HADS 8–10)	12.1	11.4	14.3	
	1 (HADS≥11)	81.7	82.7	78.6	
Depressive symptoms (HADS^b^), %	0 (HADS <8)	3.3	3.1	3.9	0.0037
0.5 (HADS 8–10)		6.8	5.7	9.7	
1 (HADS≥11)		89.9	91.2	86.4	
Psychology (Anxiety + Depressive symptoms), %	0	1.3	1.2	1.4	0.0277
0.5		2.5	2.3	3.2	
1		6.0	5.7	6.9	
1.5		13.4	12.2	16.7	
2		76.8	78.7	71.8	
Locomotion					
Tandem score (0–4), %	0 (0–3 s)	9.5	8.5	12.4	0.0072
2 (3–10 s)		6.3	5.8	7.5	
4 (≥10 s)		84.2	85.7	80.1	
Chair^d^ score (0–4), %	0 (enable)	4.8	4.3	6.2	0.0812
1 (>Q3)		20.6	19.5	23.5	
2 (Q2-Q3)		21.9	22.0	21.6	
3 (Q1-Q2)		23.5	24.4	21.1	
4 (≤Q1)		29.2	29.8	27.7	
Walking^d^ score (0–4), %	0 (enable)	1.2	1.3	1.0	0.0406
1 (>Q3)		19.3	17.8	23.6	
2 (Q2-Q3)		22.0	22.0	21.9	
3 (Q1-Q2)		27.7	28.9	24.7	
4 (≤Q1)		29.7	30.1	28.8	
PP^e^ score (0–12), mean ± SD, median (IQR)		8.7 ± 2.6	8.8 ± 2.5	8.3 ± 2.9	0.0042
		9	9	9	
Locomotion, %	0 (PP score 0–6)	18.6	16.2	25.0	<0.0001
1 (PP score 7–9)		36.9	38.4	32.9	
2 (PP score 10–12)		44.5	45.4	42.2	
Vitality					
BMI, kg/m^2^%	Underweight (<18.5)	0.3	0.4	0.2	0.5804
Normal (18.5–25)		28.9	29.1	28.5	
Overweight		45.7	46.1	44.5	
Obese (≥30)		25.1	24.4	26.9	
Grip strength, mean ± SD, median (IQR), kg		27.9 ± 10.1	28.5 ± 10.3	26.1 ± 9.4	<0.0001
		26	27	25	
Vitality (BMI + Grip strength), %	0	10.0	9.3	11.7	0.0029
0.5		21.2	19.2	25.8	
1		34.3	34.7	33.4	
1.5		27.3	28.8	23.2	
2		7.2	7.7	6.0	
Intrinsic capacity					
IC^f^ score (0–8), mean ± SD, median (IQR)		6.0 ± 1.2	6.1 ± 1.2	5.8 ± 1.4	<0.0001
		6	6	6	

Weighted data except numbers of participants, age, and sex. SD, standard deviation; IQR, interquartile; MMSE, Mini-Mental State Examination score; HADS, hospital anxiety and depression scales; Q1, Q2, Q3, quartiles; PP: physical performance; BMI, body mass index; IC: intrinsic capacity.

^a^ MMSE, possible scores range from 0 to 30, lower values indicating worse cognitive status.

^b^ HADS, anxiety and depressive symptoms range from 0 to 21, with higher values indicating worse health status.

^c^ Tandem class range from 0–4, higher class indicates best performance.

^d^ Chair and Walking scores country quartiles, class ≤ Q1 indicates best performance, class > Q3 indicates worst performance.

^e^ PP, possible scores range from 0 to 12, lower values indicate worse performance.

^f^ IC, possible scores range from 0 to 8, with 0 indicating worst status.

**FIGURE 2:**
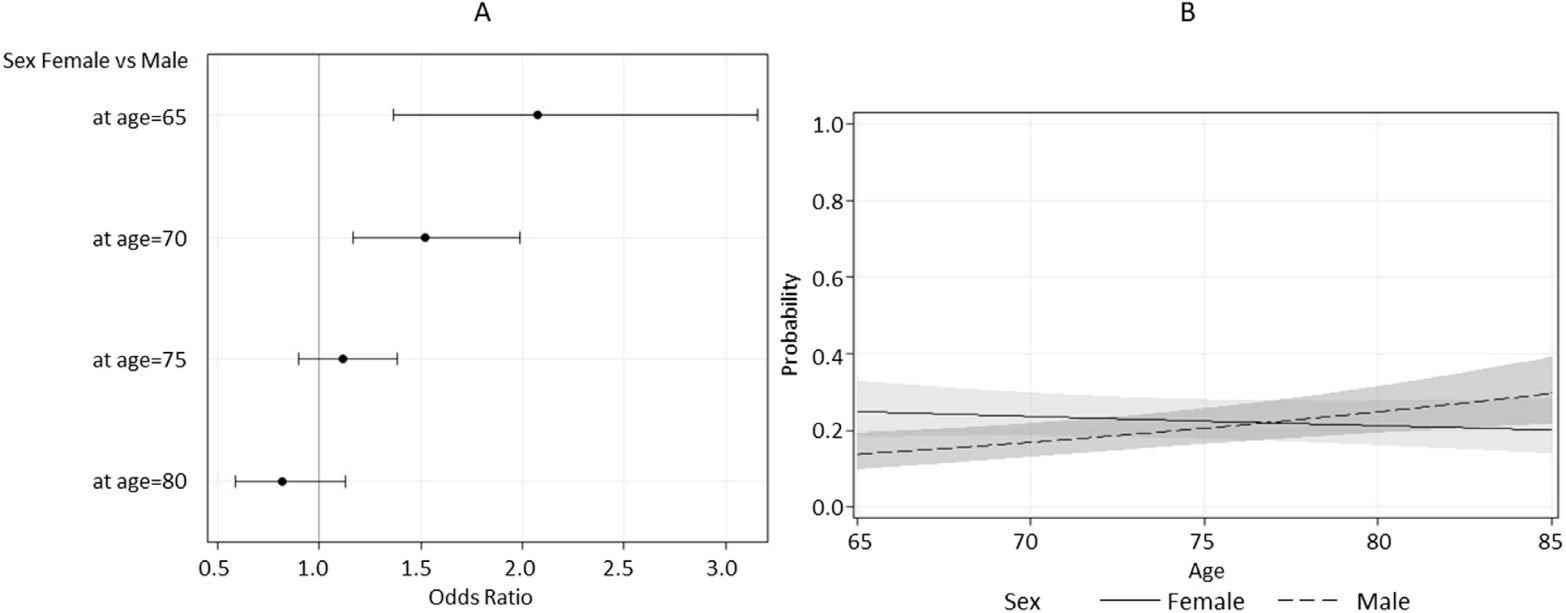
Estimated Odds Ratio **(A)** and estimated probability **(B)** for sex as a function of age, with 95% confidence limits.

According to the univariable logistic regression analysis (Table 3), adjusted for age, sex, and country, falls after 12–18 months were significantly associated with IC. Other significant predictors included tobacco use, cardiovascular disease, analgesic/anti-inflammatory medication use, clinical OA, and joint replacement. These associations remained significant in the multivariable analysis, which also identified one significant interaction between age and sex. In particular, the estimated odds ratio for a five-year increase in age differed between males and females. Among men, a five-year difference in age increases the odds of falling by almost 1.3 times, whereas among women, there is effectively no increase. In fact, women have a higher risk of falling after 12–18 months at younger ages (65–70), and this effect becomes insignificant at around the age of 73 (Figure 2). The odds of experiencing a fall during the 12–18 months follow-up were 1.5 times higher (95% CI: 1.10–2.04) for individuals with a history of hip and/or knee replacements compared to those without, after adjusting for other covariates. Similarly, the odds of falling were 1.39 times higher (95% CI: 1.05–1.82) for individuals with clinical OA of the hand only or in combination with hip and/or knee. The use of analgesic/anti-inflammatory medication was also associated with an increased risk of falls (OR = 1.31, 95% CI: 1.02–1.67). Interestingly, current tobacco consumption appeared to be a protective factor against falls. Finally, close to significance (P = 0.06) was the fall odds of individuals with cardiovascular disease (OR = 1.25, 95% CI: 0.99–1.57). Low IC (<5) was estimated to be 1.57 (95% CI: 1.19–2.08) times more likely to fall after 12–18 months.

**TABLE 3:** Univariable and Multivariable models for falls 12–18 months after baseline.

	Univariable adjusted model			Multivariable model		
	OR	95% CI	*P*	OR	95% CI	*P*
Up to elementary education	0.90	0.72–1.13	0.3522			
Marital status (Single/divorced/widowed/living apart)	1.14	0.92–1.42	0.2362			
Income			0.1203			
With great/some difficulty	1.24	0.95–1.63				
Fairly easily/Easily	1.00					
Alcohol consumption (yes)	0.83	0.64–1.08	0.1588			
Tobacco consumption			**0.0221**			0.0374
Never and In the past	1.00			1.00		
Currently	0.59	0.38–0.93		0.62	0.39–0.97	
Chronic lung disease	1.04	0.78–1.38	0.8171			
Cardiovascular disease	1.29	1.03–1.61	**0.0284**	1.25	0.99–1.57	0.0601
Peripheral artery disease	1.00	0.72–1.40	0.9831			
Diabetes mellitus	1.08	0.80–1.45	0.6314			
Stroke	1.11	0.71–1.74	0.6556			
Cancer	1.11	0.85–1.45	0.4536			
Osteoporosis	1.15	0.87–1.52	0.3298			
Clinical osteoarthritis			**0.0007**			0.0149
No	1.00			1.00		
Hip and/or knee	1.01	0.75–1.36	0.9502	0.84	0.62–1.15	0.2690
Hand and/or (hip and/or knee)	1.65	1.27–2.15	**0.0002**	1.39	1.05–1.82	0.0198
Joint replacements			**0.0008**			0.0070
No	1.00			1.00		
Lower limb	1.67	1.23–2.25	**0.0009**	1.50	1.10–2.04	0.0111
Other	0.40	0.14–1.16	0.0907	0.38	0.13–1.12	0.0783
Analgesic/Anti-inflammatory medication	1.45	1.15–1.83	**0.0015**	1.31	1.02–1.67	0.0318
Psycholeptic medication	1.12	0.79–1.59	0.5197			
IC^a^ score			**<0.0001**			0.0016
<5	1.97	1.53–2.56		1.57	1.19–2.08	
≥5	1.00					
Age units = 5 at Female sex				0.93	0.81–1.08	0.3666
Age units = 5 at Male sex				1.27	1.10–1.48	0.0012

Models adjusted for age, sex and country; multivariate model additionally adjusted for age*sex interaction (*P* = 0.0025).

OR, odds ratio; CI, confidence intervals; IC: intrinsic capacity.

^a^ IC, possible scores range from 0 to 8, with 0 indicating worst status.

## 4 Discussion

This study contributes to the growing body of research exploring IC as a multidimensional construct reflecting physical and mental reserves in older adults. Our findings suggest that lower IC, operationalized as a composite score based on cognitive, psychological, physical performance, and vitality domains, is associated with a higher risk of falls in community-dwelling older individuals. Although this result is in line with previous studies (Chew et al., 2021), it should be interpreted cautiously. The IC construct applied in this study was not based on the simplified screening model proposed by the WHO ICOPE guidelines, but rather derived from available variables in the EPOSA dataset. This approach reflects a more in-depth, albeit still partial, assessment, lacking sensory domains such as vision and hearing, and key psychometric validation. Therefore, our findings do not support or refute the predictive validity of the WHO-recommended first-stage IC assessment. Rather, they suggest that IC domains, as modeled here, can conceptually reflect vulnerability to falls, consistent with previous literature (Cacciatore et al., 2024; Muneera et al., 2023).

Our results are consistent with recent evidence from a Chinese cohort, where declines in locomotive, psychological, and sensory IC domains were associated with a markedly increased risk of falls, and cognitive decline was an additional predictor of disability (Liu et al., 2025). Taken together, these findings suggest that comprehensive assessment across several IC domains, even when operationalized differently, is critical to identifying individuals at high risk of falls.

Participants who experienced falls presented a profile characterized by multiple vulnerabilities, including cognitive deficits, increased depressive symptoms, reduced muscle strength, and mobility impairment—elements that are all reflected in the IC domains. These observations align with previous literature identifying these domains as important risk factors for falls (Cacciatore et al., 2024; Muneera et al., 2023).

Our analysis further highlighted several sociodemographic and clinical factors associated with fall risk, alongside the crucial role of IC. Participants with lower IC scores were more likely to be women (81.7%) and older (45.9% over 78 years), confirming that functional vulnerability disproportionately affects older women. Consistently, women reported a higher incidence of falls, supporting findings from large studies such as the English Longitudinal Study of Ageing, which found significantly higher fall rates in women compared to men (29.1% vs. 23.5%) (Gale et al., 2016). Additionally, gender disparities exist in healthcare-seeking behaviors related to falls, with women more likely than men to seek medical assistance (37.5% vs. 24.3%) and discuss fall prevention with healthcare providers (31.2% vs. 24.3%) (Patton et al., 2022; Stevens et al., 2012). Notably, the multivariable analysis identified a significant interaction between age and sex, indicating that the impact of aging on fall risk differs between men and women. Specifically, in women, the risk of falls tends to decrease with increasing age, whereas in men, the risk progressively increases as they grow older. This suggests that risk factors for falls may operate differently between sexes, potentially due to biological, behavioral, or social differences. It is possible that older women adopt compensatory strategies or lifestyle modifications that reduce their fall risk, or that frailer women are more likely to experience higher mortality or earlier institutionalization, leading to a selection effect. In contrast, the rising risk in men may reflect lower engagement in preventive behaviors or the progression of health conditions that contribute to falls over time. These findings highlight the importance of considering gender and age interactions when designing fall prevention interventions tailored to the specific needs of at-risk groups.

Additional insights emerged regarding the role of pharmacological interventions, chronic cardiac diseases, and surgical procedures. Our data show that previous hip and/or knee replacements, the use of analgesic/anti-inflammatory medications, and the presence of OA were significantly associated with higher fall risk. These findings are consistent with the existing literature. For instance, pain and disability often persist after total joint arthroplasty, potentially predisposing individuals to a higher risk of falls. Approximately one-third of individuals experience at least one fall in the year following total knee arthroplasty or total hip arthroplasty, with the reported fall rate after total knee arthroplasty ranging from 14.1% to 38.3% (Liu et al., 2020). Previous research also shows that patients with symptomatic knee OA suffer from joint pain, stiffness, and muscle weakness, leading to functional decline and increased fall risk compared to healthy older adults without OA symptoms (Chen et al., 2019; Thompson et al., 2017; Cuevas-Trisan, 2017). The elevated risk primarily attributed to various factors, such as knee instability, muscle weakness, and a significant decline in basic functional abilities (Hadjistavropoulos et al., 2010; Manlapaz et al., 2019). Beyond functional deterioration, psychological factors such as fear of falling also play a role, and may result from gait disturbances, postural control deficits, pain, proprioceptive dysfunction, and obesity (Rosadi et al., 2022). Regarding OA of the hand, its association with fall risk appears more controversial and less consistently documented in the literature (Wilfong et al., 2023). Unlike lower limb OA, hand OA does not directly impair locomotion or balance, suggesting that its contribution to falls might be mediated by other factors such as reduced manual dexterity or grip strength, which could indirectly affect stability and the ability to prevent or recover from falls. In our analysis, individuals with OA of the hand alone or in combination with hip and/or knee OA showed an increased fall risk, highlighting the need for further research to clarify the mechanisms underlying this association. Finally, anti-inflammatory medications may increase fall risk due to side effects including dizziness, mood changes, and confusion, especially in older adults (Hegeman et al., 2009). Although the literature on this topic remains limited and heterogeneous, several systematic reviews have concluded that NSAID use in older adults is associated with increased fall incidence (Findley and Bulloch, 2015). Altogether, these findings underscore the importance of comprehensive geriatric evaluation to balance the benefits and risks of pharmacological and surgical interventions, ideally through personalized care approaches that reduce fall risk while managing chronic conditions.

Our analysis revealed a borderline significant association between cardiovascular disease and fall risk. While not conclusive, this finding may suggest a complex interplay between chronic conditions and the aging process. Intrinsic capacity and resilience are key to maintaining physiological homeostasis in older age. However, a breakdown in resilience mechanisms—whether due to advancing age or chronic illness—may accelerate biological aging, potentially triggering or exacerbating geriatric syndromes such as falls and functional decline (de Souto Barreto et al., 2023; Promislow et al., 2022).

### 4.1 Limitations and strengths

This study has some limitations. First, its observational design does not permit causal inference. Second, the IC construct used here was not originally designed to reflect the WHO ICOPE tool and lacks the sensory domain (vision and hearing). As such, it may not fully capture the multidimensional nature of IC as conceptualized by the WHO, and its psychometric properties remain untested. Additionally, the use of a 12–18 months recall period for falls may have introduced recall bias and underreporting. Heterogeneity across participating countries—in terms of recruitment strategies and population characteristics—could also affect generalizability, even though we adjusted for country in the analyses. Moreover, we excluded physical activity measures (e.g., minutes of activity or kilocalories/week) from the models due to high proportions of missing data and the lack of significant associations in preliminary analyses. Although this decision helped preserve statistical power, it may have limited our ability to capture relevant behavioral factors associated with fall risk.

The main strengths of the study include the use of a large, multicenter European dataset, a multidimensional approach to capturing physical and mental capacities, and the inclusion of relevant clinical and sociodemographic covariates. Furthermore, the analysis of interaction terms allowed us to identify meaningful sex- and age-related differences in fall risk.

### 4.2 Conclusions

In conclusion, this study found that a lower intrinsic capacity score, derived from multiple domains, was associated with a greater likelihood of falling among older adults. However, this association was modest and based on a construct that lacks formal validation. As such, the IC score used in this study should not be interpreted as a substitute for established fall risk tools. Further research is needed to develop standardized and validated IC-based instruments, evaluate their predictive value, and determine their utility in clinical settings. Nonetheless, our findings support the conceptual relevance of IC in understanding vulnerability in aging and highlight the importance of multidimensional approaches to fall risk assessment.

## Group members of EPOSA Research Group

Nikolaus T, Peter R, Denkinger MD, Herbolsheimer F, Maggi SAUTHORID, Zambon S, Limongi FAUTHORID, Noale MAUTHORID, Siviero PAUTHORID, Deeg DJ, van der Pas S, Schaap LA, van Schoor NM, Timmermans EJ, Otero A, Castell MV, Sanchez-Martinez M, Quieipo R, Pedersen NL, Broumandi R, Dennison EM, Cooper C, Edwards MH, Parsons C.

## Data Availability

The datasets presented in this article are not readily available because Data were used under license for the current study, and so are not publicly available. Data are however available from the authors upon reasonable request and with permission of the EPOSA Research Group. Requests to access the datasets should be directed to https://www.eposa.org/.
