# Supplementary material for: Impact of a four-domain intrinsic capacity measure on falls: findings from the EPOSA study
Source: Front Aging. 2025 Sep 5;6:1645712. doi: 10.3389/fragi.2025.1645712 (PMC12447103; doi:10.3389/fragi.2025.1645712)
Supplement: Supplementary file 1 [file Table1.docx]

Supplementary Material

**Table S1.** Characteristics of participants drop-out at 12-18 months follow-up

| **Baseline Characteristics** | | | | **12-18 months Follow-up** | | ***P*** |
| --- | --- | --- | --- | --- | --- | --- |
|  | | | | **Drop-out**  (n=470) | **No Drop-out**  (n=2,127) |  |
| **Age**, mean±SD, median (IQR), years | | | | 75.1±5.6 | 73.7±5.0) | <.0001 |
|  | | | | 75 (70-80) | 74 (70-77 |  |
| **Female sex**, % | | | | 57.02 | 49.98 | 0.0057 |
| **Country**, % | | Germany | | 12.1 | 13.7 | <.0001 |
| Italy | | | | 15.0 | 15.0 |  |
| The Netherlands | | | | 19.5 | 18.2 |  |
| Spain | | | | 12.1 | 19.1 |  |
| Sweden | | | | 30.8 | 20.2 |  |
| UK | | | | 28.2 | 13.7 |  |
| **Up to elementary education**, % | | | | 57.8 | 42.5 | <.0001 |
| **Marital status** (Single/divorced/widowed/living apart), % | | | | 36.5 | 33.3 | 0.1938 |
| **Income**, % | | With great/some difficulty | | 27.9 | 16.6 | <.0001 |
| Fairly easily | | | | 49.0 | 50.3 |  |
| Easily | | | | 23.1 | 33.1 |  |
| **Alcohol consumption** (yes), % |  | | | 70.5 | 77.3 | 0.0021 |
| **Tobacco consumption**, % | Never | | | 51.7 | 49.5 | 0.0002 |
| Currently | | | | 12.8 | 7.6 |  |
| In the past | | | | 35.5 | 42.9 |  |
| **Chronic lung disease**, % | | | | 14.2 | 12.7 | 0.3829 |
| **Cardiovascular disease**, % | | | | 28.2 | 24.0 | 0.0564 |
| **Peripheral artery disease**, % | | | | 18.3 | 9.9 | <.0001 |
| **Diabetes mellitus**, % | | | | 15.8 | 11.9 | 0.0174 |
| **Stroke**, % | | | | 7.3 | 4.4 | 0.0102 |
| **Cancer**, % | | | | 13.3 | 14.1 | 0.6547 |
| **Osteoporosis**, % | | | | 22.2 | 15.3 | 0.0003 |
| **Clinical osteoarthritis**, % | | No | | 63.8 | 70.5 | 0.0156 |
| Hand | | | | 8.3 | 8.4 |  |
| Hip and/or knee | | | | 17.3 | 13.3 |  |
| Hand and (hip and/or knee) | | | | 10.7 | 7.8 |  |
| **Joint replacements**, % | | | No | 86.2 | 88.9 | 0.1115 |
| Lower limb | | | | 12.8 | 9.6 |  |
| Other | | | | 1.1 | 1.5 |  |
| **Analgesic/Anti-inflammatory medication**, % | | | | 25.7 | 24.2 | 0.5156 |
| **Psycholeptic medication**, % | | | | 14.5 | 8.8 | 0.0002 |

Weighted data except numbers of participants, age, and sex. SD, standard deviation; IQR, interquartile.

**Table S2.** Intrinsic capacity of participants drop-out at 12-18 months follow-up

| **Intrinsic capacity domains** | | | | | **12-18 months Follow-up** | | ***P*** |
| --- | --- | --- | --- | --- | --- | --- | --- |
|  | | | | | **Drop-out**  (n=470) | **No Drop-out**  (n=2,127) |  |
| **Cognition** |  | | | |  |  |  |
| **Cognition** (MMSE^#^), % | 0 (MMSE score ≤18) | | | | 3.0 | 0.5 | <.0001 |
| 1 (MMSE score 18-24) | | | | | 13.7 | 5.8 |  |
| 2 (MMSE score ≥24) | | | | | 83.3 | 93.8 |  |
| **Psychology** | | |  | |  |  |  |
| **Anxiety symptoms** (HADS^§^), % | | 0 (HADS <8) | | | 14.0 | 6.2 | <.0001 |
|  | 0.5 (HADS 8-10) | | | | 18.1 | 12.1 |  |
|  | 1 (HADS >=11) | | | | 67.9 | 81.7 |  |
| **Depressive symptoms** (HADS^§^), % | | | 0 (HADS <8) | | 5.6 | 3.3 | <.0001 |
| 0.5 (HADS 8-10) | | | | | 14.6 | 6.8 |  |
| 1 (HADS >=11) | | | | | 79.8 | 89.9 |  |
| **Psychology (Anxiety+Depressive symptoms)**, % | | | | 0 | 2.4 | 1.3 | <.0001 |
| 0.5 | | | | | 5.0 | 2.5 |  |
| 1 | | | | | 13.6 | 6.0 |  |
| 1.5 | | | | | 20.1 | 13.4 |  |
| 2 | | | | | 58.9 | 76.8 |  |
| **Locomotion** |  | | | |  |  |  |
| **Tandem**^~^ **score** (0-4), % | 0 (0-3 sec) | | | | 21.5 | 9.5 | <.0001 |
| 2 (3-10 sec) | | | | | 7.7 | 6.3 |  |
| 4 (≥10 sec) | | | | | 70.8 | 84.2 |  |
| **Chair**^‡^ **score** (0-4), % | 0 (enable) | | | | 11.4 | 4.8 | <.0001 |
| 1 (>Q3) | | | | | 24.0 | 20.6 |  |
| 2 (Q2-Q3) | | | | | 22.6 | 21.9 |  |
| 3 (Q1-Q2) | | | | | 19.2 | 23.5 |  |
| 4 (≤Q1) | | | | | 22.9 | 29.2 |  |
| **Walking**^‡^ **score** (0-4), % | 0 (enable) | | | | 2.7 | 1.2 | <.0001 |
| 1 (>Q3) | | | | | 29.1 | 19.3 |  |
| 2 (Q2-Q3) | | | | | 24.1 | 22.0 |  |
| 3 (Q1-Q2) | | | | | 28.6 | 27.7 |  |
| 4 (≤Q1) | | | | | 15.5 | 29.7 |  |
| **PP**^†^ **score** (0-12), mean±SD, median (IQR) | | | | | 7.4±3.2 | 8.7±2.6 | <.0001 |
|  | | | | | 8 (6-10) | 9 (7-11) |  |
| **Locomotion**, % | 0 (PP score 0-6) | | | | 32.2 | 18.6 | <.0001 |
| 1 (PP score 7-9) | | | | | 38.0 | 36.9 |  |
| 2 (PP score 10-12) | | | | | 29.8 | 44.5 |  |
| **Vitality** |  | | | |  |  |  |
| **BMI**, kg/m^2^ % | Underweight (<18.5) | | | | 0.9 | 0.2 | 0.0989 |
| Normal (18.5-25) | | | | | 25.4 | 28.5 |  |
| Overweight (25-30) | | | | | 45.3 | 45.7 |  |
| Obese (≥30) | | | | | 28.4 | 25.1 |  |
| **Grip strength**, mean±SD, median (IQR), kg | | | | | 25.9±10.6 | 27.9±10.1 | <.0001 |
|  | | | | | 23.5 (18-33) | 26.3 (20-35) |  |
| **Vitality (BMI+Grip strength)**, % | | | 0 | | 13.8 | 10.0 | 0.0015 |
| 0.5 | | | | | 26.2 | 21.2 |  |
| 1 | | | | | 32.2 | 34.3 |  |
| 1.5 | | | | | 23.7 | 27.3 |  |
| 2 | | | | | 4.1 | 7.2 |  |
| **Intrinsic Capacity** |  | | | |  |  |  |
| **IC^*^ score (0-8)**, mean±SD, median (IQR) | | | | | 5.3±1.5 | 6.0±1.2 | <.0001 |
|  | | | | | 5.5 (4.5-6.5) | 6.0 (5.0-7.0) |  |

Weighted data except numbers of participants, age, and sex. SD, standard deviation; IQR, interquartile; MMSE, Mini-Mental State Examination score; HADS, Hospital Anxiety and Depression Scales; Q1, Q2, Q3, quartiles; PP: Physical Performance; BMI, body mass index; IC: intrinsic capacity.

^#^ MMSE possible scores range from 0 to 30, lower values indicating worse cognitive status.

^§^ HADS anxiety and depressive symptoms range from 0 to 21, with higher values indicating worse health status.

^~^ Tandem class range from 0-4, higher class indicates best performance.

^‡^ Chair and Walking scores country quartiles**,** class ≤Q1 indicates best performance, class >Q3 indicates worst performance.

^†^ PP possible scores range from 0 to 12, lower values indicate worse performance.

^*^ IC possible scores range from 0 to 8, with 0 indicating worst status.

**Table S3.** Baseline characteristics

| **Baseline Characteristics** | **Total** | **Country** | | | | | | | | ***P*** |  |
| --- | --- | --- | --- | --- | --- | --- | --- | --- | --- | --- | --- |
|  |  | **Germany** | | **Italy** | **The Netherlands** | **Spain** | **Sweden** | **UK** | |  | |
|  | (n=2,597) | (n=368) | (n=451) | | (n=508) | (n=470) | (n=422) | | (n=378) |  | |
| **Age**, mean±SD, median (IQR), years | 74.0±5.1 | 74.3±5.0 | 73.2±5.2 | | 75.0±5.7 | 74.7±5.5 | 71.6±4.7 | | 75.1±2.6 | <.0001 | |
|  | 73 (70-78) | 73 (70-77) | 73 (69-77) | | 75 (70-80) | 74 (70-79) | 70 (68-74) | | 75 (73-77) |  | |
| **Female sex**, % | 51.3 | 41.3 | 53.2 | | 52.2 | 49.8 | 60.2 | | 49.5 | <.0001 | |
| **Up to elementary education**, % | 45.3 | 50.4 | 77.3 | | 25.0 | 70.6 | 22.1 | | 21.4 | <.0001 | |
| **Marital status** (Single/divorced/widowed/living apart), % | 33.9 | 31.9 | 27.0 | | 39.1 | 30.6 | 40.7 | | 32.8 | <.0001 | |
| **Income**, % |  |  |  | |  |  |  | |  |  | |
| With great/some difficulty | 18.7 | 3.5 | 42.4 | | 11.4 | 29.9 | 5.5 | | 14.9 |  | |
| Fairly easily | 50.1 | 53.6 | 54.5 | | 35.6 | 58.1 | 48.6 | | 53.6 |  | |
| Easily | 31.3 | 42.9 | 3.2 | | 53.0 | 11.9 | 45.9 | | 31.6 |  | |
| **Alcohol consumption** (yes), % | 76.0 | 89.4 | 78.7 | | 82.1 | 40.1 | 90.6 | | 80.2 | <.0001 | |
| **Tobacco consumption**, % |  |  |  | |  |  |  | |  |  | |
| Never | 49.9 | 52.4 | 58.4 | | 37.3 | 59.2 | 41.9 | | 53.3 | <.0001 | |
| Currently | 8.6 | 6.1 | 8.2 | | 10.5 | 10.0 | 10.9 | | 3.0 |  | |
| In the past | 41.5 | 41.5 | 33.4 | | 52.2 | 30.8 | 47.2 | | 43.6 |  | |
| **Chronic lung disease**, % | 13.0 | 15.4 | 7.1 | | 12.4 | 19.9 | 11.0 | | 12.1 | <.0001 | |
| **Cardiovascular disease**, % | 24.8 | 21.0 | 23.9 | | 27.8 | 31.0 | 22.6 | | 19.1 | 0.0008 | |
| **Peripheral artery disease**, % | 11.4 | 13.3 | 12.2 | | 19.6 | 11.1 | 7.3 | | 1.5 | <.0001 | |
| **Diabetes mellitus**, % | 12.5 | 10.8 | 12.0 | | 15.3 | 17.0 | 6.7 | | 12.7 | <.0001 | |
| **Stroke**, % | 5.0 | 3.3 | 4.4 | | 5.7 | 5.7 | 4.9 | | 5.5 | 0.6013 | |
| **Cancer**, % | 14.0 | 18.9 | 14.1 | | 13.4 | 9.7 | 13.4 | | 16.5 | 0.0079 | |
| **Osteoporosis**, % | 16.5 | 10.6 | 20.3 | | 18.4 | 22.2 | 7.8 | | 18.9 | <.0001 | |
| **Clinical osteoarthritis**, % |  |  |  | |  |  |  | |  |  | |
| No | 69.3 | 79.0 | 56.6 | | 75.6 | 66.5 | 67.7 | | 73.6 | <.0001 | |
| Hand | 8.3 | 9.5 | 9.2 | | 3.9 | 8.0 | 11.8 | | 8.3 |  | |
| Hip and/or knee | 14.1 | 8.4 | 19.9 | | 14.0 | 15.4 | 11.9 | | 12.9 |  | |
| Hand and (hip and/or knee) | 8.3 | 3.1 | 14.3 | | 6.5 | 10.1 | 8.6 | | 5.3 |  | |
| **Joint replacements**, % |  |  |  | |  |  |  | |  |  | |
| No | 88.4 | 88.2 | 89.4 | | 87.0 | 89.6 | 88.1 | | 88.0 | 0.0690 | |
| Lower limb | 10.2 | 11.4 | 10.4 | | 11.4 | 8.0 | 9.6 | | 10.9 |  | |
| Other | 1.4 | 0.4 | 0.2 | | 1.6 | 2.4 | 2.3 | | 1.01 |  | |
| **Analgesic/Anti-inflammatory medication**, % | 24.5 | 15.3 | 22.7 | | 14.2 | 43.1 | 16.3 | | 38.4 | <.0001 | |
| **Psycholeptic medication, %** | 9.9 | 0.9 | 14.3 | | 6.1 | 22.0 | 7.7 | | 4.1 | <.0001 | |

Weighted data except numbers of participants, age, and sex. SD, standard deviation; IQR, interquartile.

**Table S4.** Baseline Intrinsic Capacity domains

| **Intrinsic Capacity domains** | **Total** | **Country** | | | | | | ***P*** |
| --- | --- | --- | --- | --- | --- | --- | --- | --- |
|  |  | **Germany** | **Italy** | **The Netherlands** | **Spain** | **Sweden** | **UK** |  |
|  | (n=2,597) | (n=368) | (n=451) | (n=508) | (n=470) | (n=422) | (n=378) |  |
| **Cognition** |  |  |  |  |  |  |  |  |
| **Cognition (**MMSE^#^), % |  |  |  |  |  |  |  | <.0001 |
| 0 (MMSE score ≤18) | 0.9 | 0 | 1.9 | 0.6 | 2.5 | 0 | 0.2 |  |
| 1 (MMSE score 18-24) | 7.2 | 0.4 | 16.2 | 3.1 | 9.9 | 4.7 | 8.2 |  |
| 2 (MMSE score ≥24) | 91.8 | 99.6 | 81.9 | 96.4 | 87.7 | 95.3 | 91.6 |  |
| **Psychology** |  |  |  |  |  |  |  |  |
| **Anxiety symptoms** (HADS^§^), % |  |  |  |  |  |  |  | <.0001 |
| 0 (HADS <8) | 7.7 | 4.0 | 23.2 | 3.4 | 6.5 | 2.5 | 5.2 |  |
| 0.5 (HADS 8-10) | 13.3 | 11.2 | 29.4 | 12.1 | 9.8 | 4.7 | 11.5 |  |
| 1 (HADS ≥11) | 79.1 | 84.8 | 47.3 | 84.5 | 83.7 | 92.8 | 83.3 |  |
| **Depressive symptoms** (HADS^§^) % |  |  |  |  |  |  |  | <.0001 |
| 0 (HADS <8) | 3.7 | 1.7 | 6.7 | 2.1 | 8.6 | 0.4 | 1.9 |  |
| 0.5 (HADS 8-10) | 8.2 | 6.9 | 14.1 | 10.5 | 10.4 | 0.9 | 4.9 |  |
| 1 (HADS ≥11) | 88.0 | 91.5 | 79.3 | 87.4 | 81.0 | 98.8 | 93.2 |  |
| **Psychology (Anxiety+Depressive symptoms)**, % |  |  |  |  |  |  |  |  |
| 0 | 1.5 | 0 | 4.3 | 0.6 | 2.4 | 0 | 1.0 | <.0001 |
| 0.5 | 3.0 | 2.1 | 6.4 | 1.9 | 4.3 | 0.7 | 2.0 |  |
| 1 | 7.4 | 6.2 | 18.4 | 5.2 | 7.3 | 2.1 | 4.3 |  |
| 1.5 | 14.6 | 10.8 | 30.1 | 15.3 | 13.2 | 4.8 | 11.7 |  |
| 2 | 73.5 | 80.9 | 40.8 | 77.1 | 72.8 | 92.3 | 81.0 |  |
| **Locomotion** |  |  |  |  |  |  |  |  |
| **Tandem**^~^  **score** (0-4), % |  |  |  |  |  |  |  | <.0001 |
| 0 (0-3 sec) | 11.7 | 11.1 | 13.7 | 16.5 | 10.9 | 5.3 | 12.6 |  |
| 2 (3-10 sec) | 6.5 | 0.0 | 11.9 | 6.4 | 7.0 | 2.6 | 11.2 |  |
| 4 (≥10 sec) | 81.7 | 88.9 | 74.4 | 77.1 | 82.1 | 92.1 | 76.2 |  |
| **Chair**^‡^ **score** (0-4), % |  |  |  |  |  |  |  | <.0001 |
| 0 (enable) | 6.1 | 3.9 | 6.5 | 7.5 | 5.3 | 2.9 | 11.5 |  |
| 1 (>Q3) | 21.2 | 15.1 | 22.6 | 19.6 | 22.5 | 24.2 | 22.1 |  |
| 2 (Q2-Q3) | 22.0 | 24.6 | 22.7 | 16.9 | 22.9 | 23.9 | 22.3 |  |
| 3 (Q1-Q2) | 22.7 | 15.8 | 23.7 | 22.4 | 25.3 | 24.7 | 22.4 |  |
| 4 (≤Q1) | 28.1 | 40.6 | 24.5 | 33.7 | 24.1 | 24.3 | 21.8 |  |
| **Walking**^‡^ **score** (0-4), % |  |  |  |  |  |  |  | <.0001 |
| 0 (enable) | 1.5 | 0 | 0.3 | 2.2 | 0.5 | 1.2 | 5.6 |  |
| 1 (>Q3) | 21.2 | 24.3 | 22.8 | 17.3 | 22.9 | 18.4 | 23.3 |  |
| 2 (Q2-Q3) | 22.4 | 24.2 | 25.3 | 18.4 | 23.0 | 21.3 | 23.2 |  |
| 3 (Q1-Q2) | 27.9 | 25.7 | 26.0 | 36.0 | 28.3 | 26.8 | 21.5 |  |
| 4 (≤Q1) | 27.1 | 25.9 | 26.1 | 26.1 | 25.3 | 32.4 | 26.5 |  |
| **PP**^†^ **score** (0-12), mean±SD | 8.4±2.8 | 8.8±2.6 | 8.1±2.8 | 8.4±3.0 | 8.4±2.9 | 8.9±2.5 | 7.9±2.6 | <.0001 |
| median (IQR) | 9 (7-11) | 9 (7-11) | 9 (6-10) | 9 (7-11) | 9 (7-11) | 9 (8-11) | 8 (6-10) |  |
| **Locomotion**, % |  |  |  |  |  |  |  | <.0001 |
| 0 (PP score 0-6) | 21.1 | 16.6 | 26.1 | 21.5 | 22.5 | 12.9 | 28.1 |  |
| 1 (PP score 7-9) | 37.1 | 34.7 | 35.9 | 33.6 | 35.5 | 44.4 | 39.1 |  |
| 2 (PP score 10-12) | 41.8 | 48.8 | 38.0 | 44.9 | 42.0 | 42.7 | 32.8 |  |
| **Vitality** |  |  |  |  |  |  |  |  |
| **BMI**, kg/m^2^ % |  |  |  |  |  |  |  | <.0001 |
| Underweight (<18.5) | 0.5 | 0.7 | 0.6 | 0.4 | 0.4 | 0.5 | 0.2 |  |
| Normal (18.5-25) | 28.3 | 28.9 | 24.3 | 30.6 | 21.1 | 39.9 | 23.2 |  |
| Overweight (25-30) | 45.6 | 45.1 | 48.9 | 44.3 | 46.3 | 43.1 | 46.2 |  |
| Obese (≥30) | 25.7 | 25.4 | 26.3 | 24.8 | 32.3 | 16.5 | 30.4 |  |
| **Grip strength**^†^, mean±SD, kg | 27.5±10.3 | 28.4±10.2 | 29.2±10.3 | 29.1±10.4 | 23.7±9.5 | 28.3±10.9 | 26.0±8.7 | <.0001 |
| median (IQR), | 26 (20-35) | 26.5 (20-37) | 27 (22-36) | 26 (21-38) | 22 (17-30)) | 27 (21-35.5) | 26 (19-34) |  |
| **Vitality (BMI+Grip strength)**, % |  |  |  |  |  |  |  | <.0001 |
| 0 | 10.7 | 9.1 | 9.4 | 9.1 | 18.1 | 6.0 | 12.7 |  |
| 0.5 | 22.1 | 21.0 | 20.6 | 19.6 | 28.8 | 16.4 | 28.3 |  |
| 1 | 33.9 | 30.6 | 35.5 | 34.6 | 33.8 | 37.5 | 29.2 |  |
| 1.5 | 26.6 | 32.8 | 28.8 | 27.4 | 16.6 | 30.5 | 24.8 |  |
| 2 | 6.7 | 6.4 | 5.8 | 9.4 | 2.7 | 9.7 | 5.1 |  |
| **Intrinsic capacity** |  |  |  |  |  |  |  |  |
| **IC^*^ score** (0-8), mean±SD, | 5.9±1.3 | 6.2±1.1 | 5.4±1.5 | 6.1±1.3 | 5.6±1.4 | 6.3±1.1 | 5.7±1.1 | <.0001 |
| median (IQR) | 6.0 (5.0-7.0) | 6.5 (5.5-7.0) | 5.5 (4.5-6.5) | 6.5 (5.0-7.0.0) | 5.5 (4.5-7.0) | 6.5 (5.5-7.0) | 6.0 (5.0-6.5) |  |

Weighted data except numbers of participants, age, and sex. SD, standard deviation; IQR, interquartile, MMSE, Mini-Mental State Examination score; HADS, Hospital Anxiety and Depression Scales; Q1, Q2, Q3, quartiles; PP: Physical Performance; BMI, body mass index; IC: intrinsic capacity.

^#^ MMSE possible scores range from 0 to 30, lower values indicating worse cognitive status.

^§^ HADS anxiety and depressive symptoms range from 0 to 21, with higher values indicating worse health status.

^~^ Tandem class range from 0-4, higher class indicates best performance.

^‡^ Chair and Walking scores country quartiles**,** class ≤Q1 indicates best performance, class >Q3 indicates worst performance.

^†^ PP possible scores range from 0 to 12, lower values indicate worse performance.

^*^ IC possible scores range from 0 to 8, with 0 indicating worst status.
